# Bruxism Decoded: A Case Report Revealing the Invisible Signs

**DOI:** 10.7759/cureus.88014

**Published:** 2025-07-15

**Authors:** Marta Canas Miranda, Raquel Monteiro, Catarina Afonso, Sofia Ferreira de Almeida

**Affiliations:** 1 Family Medicine, Unidade Saúde Familiar (USF) Tornada, Unidade Local de Saúde do Oeste, Caldas da Rainha, PRT; 2 Psychiatry, Wigan Child and Adolescent Mental Health Services (CAMHS), Greater Manchester Mental Health NHS Foundation Trust, Wigan, GBR

**Keywords:** anxiety, awake bruxism, bruxism, headaches, muscle relaxants, shoulder pain, sleep bruxism, tiredness

## Abstract

Bruxism is characterized by involuntary movements involving teeth grinding or clenching. It is perceived as a parafunctional activity, and it can lead to instability and various consequences such as dental wear, musculoskeletal pain, masseter hypertrophy, persistent fatigue, and tension headaches. According to the time of occurrence, it can be classified as awake bruxism and/or sleep bruxism.

This case report describes a 19-year-old woman, who presented to her family doctor with shoulder pain, headaches, and persistent tiredness despite apparent adequate sleep. She reported no other symptoms and denied any trauma or physical exertion. Her past medical history included anxiety disorder, treated with a selective serotonin reuptake inhibitor (SSRI), and irregular follow-ups with psychology and psychiatry throughout the years. The only relevant findings on the physical examination were cervical and shoulder tenderness, without functional impairment, along with noticeable wear on dental surfaces, consistent with bruxism. Further history of overall behaviour was compatible with both sleep and awake bruxism, exacerbated during periods of increased anxiety. The SSRI dose was increased, and a muscle relaxant was prescribed. A referral was made to both psychology and dentistry. After two weeks, a reduction in anxiety, headaches, and musculoskeletal pain was observed, with only sporadic use of the muscle relaxant. A dental splint was prescribed following dental evaluation, resulting in significant clinical improvement: better anxiety control, improved sleep quality, occasional musculoskeletal complaints, and reduced headaches.

Although the patient did not initially complain of bruxism, the presence of rhythmic masticatory muscle activity with repetitive contractions and increased muscle tension highlighted the need to identify this condition as a clinical target for intervention and treatment.

Early diagnosis is essential to minimize complications and prevent symptom dispersion. Otherwise, bruxism may be overlooked as a central diagnosis, and treatment opportunities may be missed. Given its multifactorial aetiology, management must be multidisciplinary, as demonstrated in this case.

## Introduction

Bruxism is a parafunctional activity characterized by repetitive, involuntary masticatory muscle movements, including clenching or grinding of the teeth and/or bracing or thrusting of the mandible [1]. It can occur while awake (awake bruxism) or asleep (sleep bruxism). Particularly, sleep bruxism is classified by the American Academy of Sleep Medicine as a movement disorder related to sleep [2].

Epidemiological studies estimate that bruxism affects approximately 8-31% of the population in general, with sleep bruxism being more prevalent in children and adolescents, while awake bruxism tends to emerge in early adulthood [3]. A systematic review on the prevalence of bruxism in adults indicates that sleep bruxism decreases with age, being more frequent among young adults and less common in older individuals [3,4]. Additionally, research on adult bruxism epidemiology reveals significant variability in prevalence estimates, largely influenced by reliance on self-reported data, which can contribute to underreporting or misdiagnosis [5].

The peak incidence often occurs between the ages of 20 and 40 years old [4]. However, diagnostic confirmation may be delayed due to its subclinical presentation and symptom overlap with other conditions.

Bruxism is frequently associated with multiple signs and symptoms, such as abnormal tooth wear, jaw or facial pain, masseter hypertrophy, temporomandibular joint discomfort, tension-type headaches, neck and back pain, and tiredness. It is also closely linked to mental health conditions, such as anxiety, stress, and certain sleep disorders [6,7].

Considering its multifactorial aetiology (biological, psychological, and exogenous factors), early recognition and a multidisciplinary approach are crucial to mitigate long-term complications and improve patient outcomes [8].

## Case presentation

A 19-year-old Caucasian woman presented to her family doctor complaining of right shoulder pain with some functional limitation, headaches, and tiredness. Her prior medical history included a depressive disorder that emerged after a major family event, resulting in hospitalization at age three due to food refusal. She also had a diagnosis of generalized anxiety disorder, with symptoms that flared in response to specific triggers, though no panic attacks had occurred in the past two years. On initial presentation, the patient was prescribed fluoxetine 20 mg daily, combined oral contraceptives, and attended psychology appointments occasionally. She denied tobacco, alcohol, or drug use.

The anamnesis revealed intense, pressure-type headaches localized frontally and occipitally, without associated symptoms such as nausea, dizziness, or visual disturbances. Headaches were exacerbated by anxiety and stressful situations but not related to work. She reported feeling tired upon waking and going to bed but denied sleep disturbances. Her chief complaint was about right shoulder pain that had persisted for several days without trauma or extreme exertion, but with radiation to the cervical region. The pain worsened with exertion and was present at night and on awakening. There was no muscle weakness or sensory alteration. She used paracetamol sporadically with slight relief.

**Figure 1:**
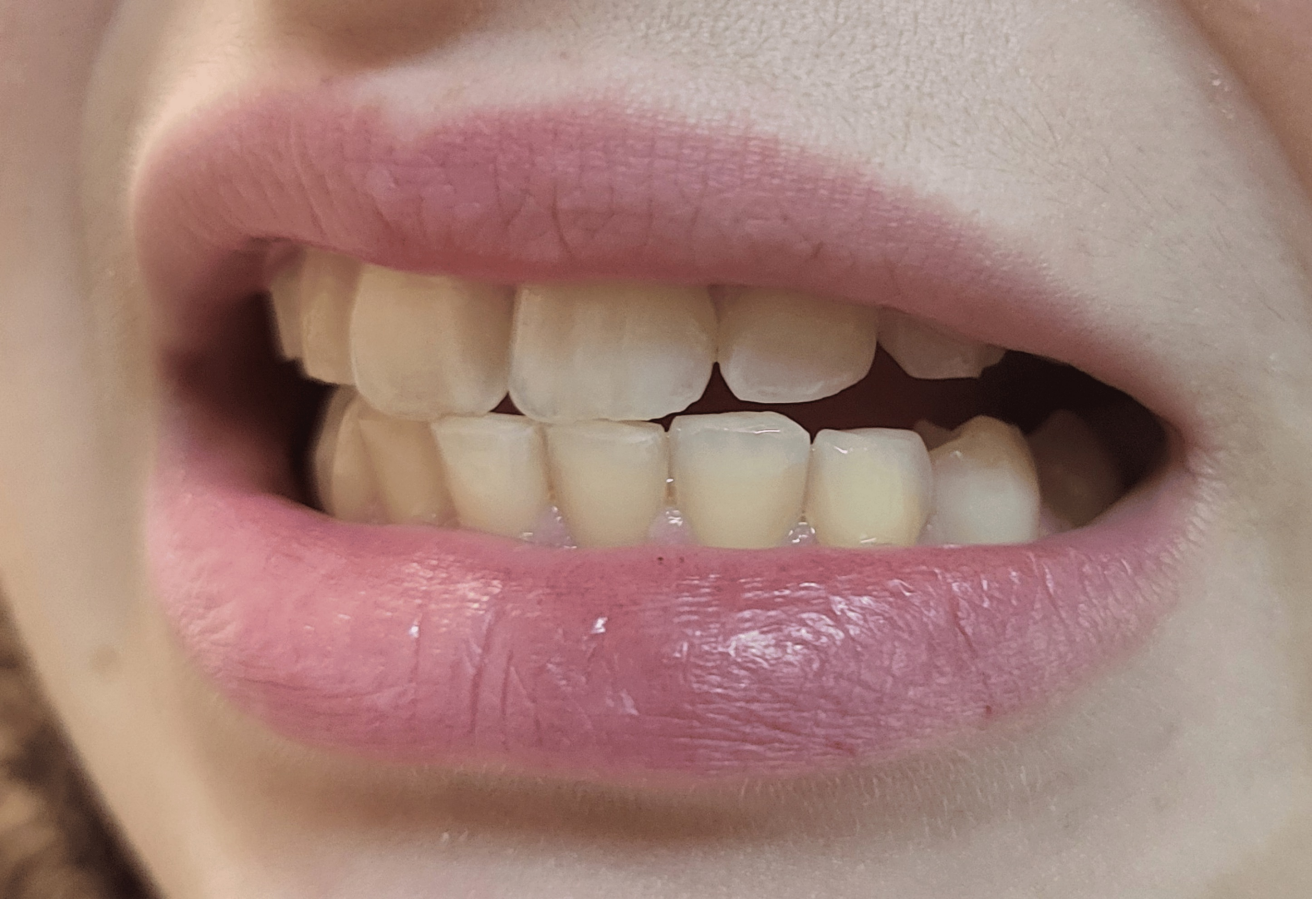
Abnormal tooth wear Dental examination shows surface wear, most notably the loss of pointed profile in all canines.

On examination, she appeared to be a healthy young woman and in a euthymic mood, though muscle tension was noted during speech. Her skin and mucosa were hydrated and without any lesions. Cardiopulmonary auscultation was normal, and neurological examination was unremarkable. Blood pressure was 104/75 mmHg, pulse 75 beats per minute, and weight 65 kg (BMI was 26.7). Cervical muscle palpation disclosed paravertebral muscle tenderness with bilateral contracture, worse on the right side. The right shoulder also showed tenderness in the suprascapular and proximal deltoid muscles, with preserved range of motion with passive movements and mild pain on active resisted movements, in particular during abduction and external rotation. Dental inspection revealed significant wear on both dental arches consistent with bruxism (Figure 1). Upon this physical finding, the patient acknowledged long-standing bruxism (both awake and during sleep) and reported recent orthodontic treatment with current use of retainers, but denied the use of a night guard.

For diagnostic purposes, laboratory tests were requested, including full blood count, renal and liver function, thyroid profile, urinalysis, fasting glucose, electrolytes and nutritional markers, and routine serologies, as well as an electrocardiogram.

Suspecting bruxism-related headache, tiredness, and shoulder pain, the treatment plan included increasing fluoxetine to 40 mg/day, prescribing analgesia and muscle relaxants for pain management, local heat application for muscle relaxation, and dental referral for the evaluation of occlusal splint use. A referral to primary care psychology was also made for anxiety and eventually adjustment disorder management (as she was starting her working life).

After six weeks, follow-up laboratory tests revealed isolated low ferritin (as seen in Table 1) without anaemia, with all other results being unremarkable. The patient reported symptomatic improvement in anxiety and musculoskeletal complaints, with minimal need for muscle relaxants, and a decreased headache frequency. Iron supplements were initiated, and she was still awaiting dental and psychological interventions.

**Table 1:** Laboratory test results All parameters tested were within the normal reference range value, with the exception of ferritin, without abnormal serum iron or hemoglobin. Laboratory values were obtained from a specific diagnostic laboratory (Portugal), which uses the reference ranges mentioned. All tests were performed at the same time and in the same lab. RBC: red blood cell; Hb: hemoglobin; Hct: hematocrit; MCV: mean corpuscular volume; WBC: white blood cell; Neutr: neutrophils; Eosi: eosinophils; Basop: basophils; Lymph: lymphocytes; Mono: monocytes; ALP: alkaline phosphatase; ALT: alanine aminotransferase; AST: aspartate aminotransferase; Creat: creatinine; FT4: free thyroxine; GGT: gamma-glutamyl transferase; HBsAb: hepatitis B surface antibody; HDL: high-density lipoprotein; HIV 1/2: human immunodeficiency virus antibody for type 1 and/or type 2; T3: triiodothyronine; TSH: thyroid-stimulating hormone; VDRL/RPR: venereal disease research laboratory/rapid plasma reagin (non-treponemal tests for syphilis diagnosis); Bilir: bilirubin; Bld: blood; Gravity: specific gravity; Gluc: glucose; Ket: ketone; Leuc: leukocytes; Nit: nitrite; Prot: protein; Urob: urobilinogen

Laboratory test	Result	Unit	Normal reference range
Hematology			
Hb	13.30	g/dL	12.00-16.00
RBC count	4.73	×10^12^/L	3.85-5.20
Hct	0.40	L/L	0.35-0.46
MCV	84.10	fL	80.00-99.00
WBC count	8.80	×10^9^/L	4.00-10.00
Neutr	6.18	×10^9^/L	1.50-8.00
Eosi	0.16	×10^9^/L	0.00-0.50
Basop	0.07	×10^9^/L	0.00-0.30
Lymph	2.07	×10^9^/L	0.80-4.00
Mono	0.32	×10^9^/L	0.00-1.20
Platelet count	339	×10^9^/L	140-440
Biochemistry			
Glucose (fasting)	78	mg/dL	70-110
Urea	26	mg/dL	19-49
Creat	0.66	mg/dL	0.50-1.10
Uric acid	4.30	mg/dL	3.10-7.80
ALT	17	U/L	<34
AST	15	U/L	10-49
GGT	10	U/L	<38
ALP	83	U/L	45-129
Total cholesterol	174	mg/dL	<190
HDL cholesterol	58	mg/dL	>65
Triglycerides	124	mg/dL	<150
Sodium	138	mmol/L	132-146
Potassium	4.30	mmol/L	3.50-5.50
Serum iron	80	μg/dL	50-170
Ferritin	17	ng/mL	35-291
Folic acid	5.90	ng/mL	>5.40
Vitamin B12	549	pg/mL	211-911
TSH	2.154	mIU/L	0.550-4.783
FT4	1.28	ng/mL	0.80-1.76
Total T3	152	ng/mL	60-181
VDRL/RPR	Not reactive		
HBsAb	<3.1	mIU/mL	Positive if >10
HIV antibody for types 1 and 2	Negative		
Urinalysis			
pH	6.50	-	4.60-8.00
Gravity	1.027		
Gluc	Negative		
Bilir	Negative		
Ket	Negative		
Prot	Negative		
Urob	0.2	mg/dL	0.2-1.0
Bld	Negative		
Nit	Negative		
Leuc	Negative		

After a month, the patient had been using a dental splint for one week and reported that her shoulder pain and headaches had subsided. She was no longer using pain relief medication. Psychological symptoms also improved with less irritability, anxiety, and muscle tension. The patient also described lighter, fragmented sleep with nocturnal awakenings and restlessness, which she was unsure had been present previously, as she may not have noticed them due to the overwhelming nature of her other symptoms. Clonazepam was initiated at a dose of 0.5 mg nightly.

A further follow-up took place a month later where the patient reported significant improvement in anxiety, sleep, and musculoskeletal symptoms, with no need for muscle relaxants. Due to service overload, the patient was still awaiting psychological support.

## Discussion

Bruxism is a complex condition with a multifactorial aetiology that involves an interrelation of biological, psychological, and behavioural factors [8]. The presented case highlights the interplay between anxiety disorders, bruxism, and musculoskeletal involvement, which is consistent with the evidence suggesting that psychological stress and anxiety exacerbate or trigger parafunctional masticatory muscle activity [7]. The patient's history of anxiety, along with documented muscle pain, fatigue, and tension headaches, reflects common comorbidities frequently observed in individuals with bruxism [6].

The identification of both awake and sleep bruxism in this young adult, initially undiagnosed, highlights the importance of thorough clinical examination, including dental assessment [1]. Dental wear patterns, masseter hypertrophy, cervical muscle contractures, and the patient's clinical history remain essential for diagnostic hypothesis, especially as bruxism is often underreported due to its involuntary nature and lack of awareness by the patient [5].

As its multifactorial aetiology, management must be multidisciplinary. In this case, pharmacological adjustment (increase of fluoxetine dose), use of muscle relaxants, referral to dental care for occlusal splint therapy, and psychological support contributed to symptom improvement [9]. The use of occlusal splints is widely accepted for reducing tooth wear and protecting dental structures, though their effect on muscle activity and anxiety is variable [8]. Psychotherapeutic interventions remain fundamental, especially given the close association between bruxism and anxiety disorders [7].

The patient's favourable response after the initiation of occlusal splint therapy and psychiatric medication adjustment supports the evidence advocating integrated multidisciplinary treatment [9].

## Conclusions

This case illustrates the critical need for early diagnosis and a comprehensive, multidisciplinary approach (coordinating between primary care, dentistry, psychiatry, and psychology) in managing bruxism, especially when associated with anxiety and musculoskeletal symptoms.

Overall, early recognition of both awake and sleep bruxism and prompt intervention are key to preventing long-lasting complications, such as temporomandibular joint disorders, chronic pain, and, consequently, diminished quality of life.

The occurrence of bruxism fluctuates across the lifespan, with higher rates in younger adults and lower rates in the elderly. These insights highlight the importance of including bruxism in differential diagnoses, particularly in patients aged 20-40, to promote timely and accurate identification and management.

Further research is needed to refine therapeutic protocols and enhance the understanding of bruxism's complex pathophysiology.
